# Randomized Comparison of Magnetic Resonance Imaging Versus Transurethral Resection for Staging New Bladder Cancers: Results From the Prospective BladderPath Trial

**DOI:** 10.1200/JCO.23.02398

**Published:** 2025-01-14

**Authors:** Richard T. Bryan, Wenyu Liu, Sarah J. Pirrie, Rashid Amir, Jean Gallagher, Ana I. Hughes, Kieran P. Jefferson, Allen Knight, Veronica Nanton, Harriet P. Mintz, Ann M. Pope, Jacob Cherian, Kingsley Ekwueme, Lyndon Gommersall, Giles Hellawell, Paul Hunter-Campbell, Gokul Kanda Swamy, Sanjeev Kotwal, Vivekanandan Kumar, David Mak, Amar Mohee, Thiagarajan Nambirajan, Douglas G. Ward, Steven J. Kennish, James W.F. Catto, Prashant Patel, Nicholas D. James, BladderPath Collaborative Group

**Affiliations:** ^1^ Bladder Cancer Research Centre, Department of Cancer and Genomic Sciences, University of Birmingham, Birmingham, United Kingdom; ^2^ The Translational Epidemiology Unit, Nuffield Department of Population Health, University of Oxford, Oxford, United Kingdom; ^3^ Cancer Research UK Clinical Trials Unit, University of Birmingham, Birmingham, United Kingdom; ^4^ University Hospitals Birmingham NHS Foundation Trust, Birmingham, United Kingdom; ^5^ Patient Representative, Tetbury, United Kingdom; ^6^ University Hospital Coventry and Warwickshire, Coventry, United Kingdom; ^7^ Action Bladder Cancer, United Kingdom; ^8^ Warwick Medical School, University of Warwick, Coventry, United Kingdom; ^9^ MRC Clinical Trials Unit, UCL, London, United Kingdom; ^10^ The Royal Oldham Hospital, Northern Care Alliance NHS Foundation Trust, Oldham, United Kingdom; ^11^ Betsi Cadwaladr University Health Board—Glan Clwyd Hospital, Rhyl, United Kingdom; ^12^ University Hospitals of North Midlands—Royal Stoke Hospital, Stoke-on-Trent, United Kingdom; ^13^ London North West University Healthcare NHS Trust—Northwick Park Hospital, London, United Kingdom; ^14^ University Hospitals Plymouth NHS Trust—Derriford Hospital, Plymouth, United Kingdom; ^15^ Swansea Bay University Health Board—Morriston Hospital, Swansea, United Kingdom; ^16^ Leeds Teaching Hospitals NHS Trust—St James' University Hospital, Leeds, United Kingdom; ^17^ Norfolk and Norwich University Hospitals NHS Foundation Trust, United Kingdom; ^18^ The Royal Wolverhampton NHS Trust—New Cross Hospital, Wolverhampton, United Kingdom; ^19^ Manchester University Hospitals NHS Foundation Trust, Manchester, United Kingdom; ^20^ Wirral University Teaching Hospital NHS Foundation Trust—Arrowe Park Hospital, Birkenhead, United Kingdom; ^21^ Sheffield Teaching Hospitals NHS Trust, Sheffield, United Kingdom; ^22^ Division of Clinical Medicine, School of Medicine & Population Health, University of Sheffield, Sheffield, United Kingdom; ^23^ University College London Hospitals NHS Foundation Trust, London, United Kingdom; ^24^ Institute of Cancer Research, London, United Kingdom; ^25^ The Royal Marsden NHS Foundation Trust, London, United Kingdom

## Abstract

**PURPOSE:**

Transurethral resection of bladder tumor (TURBT) is the initial staging procedure for new bladder cancers (BCs). For muscle-invasive bladder cancers (MIBCs), TURBT may delay definitive treatment. We investigated whether definitive treatment can be expedited for MIBC using flexible cystoscopic biopsy and multiparametric magnetic resonance imaging (mpMRI) for initial staging.

**PATIENTS AND METHODS:**

We conducted a prospective open-label, randomized study conducted within 17 UK hospitals (registered as ISRCTN 35296862). Participants with suspected new BC were randomly assigned 1:1 to TURBT-staged or mpMRI-staged care, with minimization factors of sex, age, and clinician visual assessment of stage. Blinding was not possible. Patients unable/unwilling to undergo mpMRI or with previous BC were ineligible. The study had two stages with separate primary outcomes of feasibility and time to correct treatment (TTCT) for MIBC, respectively.

**RESULTS:**

Between May 31, 2018, and December 31, 2021, 638 patients were screened, and 143 participants randomly assigned to TURBT (n = 72; 55 males, 15 MIBCs) or initial mpMRI (n = 71; 53 males, 14 MIBCs). For feasibility, 36 of 39 (92% [95% CI, 79 to 98]) participants with suspected MIBC underwent mpMRI. The median TTCT for participants with MIBC was significantly shorter with initial mpMRI (n = 12, 53 days [95% CI, 20 to 89] *v* n = 14, 98 days [95% CI, 72 to 125] for TURBT, log-rank *P* .02). There was no detriment for participants with non-MIBC (median TTCT: n = 30, 17 days [95% CI, 8 to 25] for mpMRI *v* n = 28, 14 days [95% CI, 10 to 29] for TURBT, log-rank *P* = .67). No serious adverse events were reported.

**CONCLUSION:**

The mpMRI-directed pathway led to a 45-day reduction in TTCT for MIBC. Incorporating mpMRI ahead of TURBT into the standard pathway was beneficial for all patients with suspected MIBC.

## INTRODUCTION

Bladder cancer (BC) is a common malignancy with high health care costs.^1-3^ Most patients (75%-80%) present with non–muscle-invasive bladder cancer (NMIBC, stages Ta/T1/Tis),^4-6^ and the remainder with muscle-invasive bladder cancer (MIBC, stages T2-4). MIBC is an aggressive tumor with 5-year survival rates of 27%-50% despite radical treatment.^4^ Standard management for patients with BC commences with transurethral resection of bladder tumor (TURBT),^6^ established over 100 years ago.^7^ Although TURBT is the treatment of choice for NMIBC,^6^ for MIBC, it is a diagnostic procedure that is followed by definitive treatment.^4,6,8^ TURBT is generally well tolerated, although complications include overt or asymptomatic bladder perforation (2%-58% of patients),^9-11^ readmission within 30-days (4%-5%),^12,13^ and reoperation (2%)^13^; 30-day mortality is 1%^13^ and post-TURBT tumor dissemination is possible.^14,15^ TURBT may understage high-grade cancers (30%-46% of stage T1 patients undergoing radical cystectomy are subsequently upstaged to MIBC).^16-18^ After TURBT, accurate radiologic staging by computed tomography (CT) or magnetic resonance imaging (MRI) can be impeded by artifacts (perivesical inflammation and reactive lymph nodes masquerading as tumor extension and metastases, respectively^19,20^).

**CONTEXT**

**Key Objective**
To investigate whether patients with muscle-invasive bladder cancer (MIBC) can be expedited to definitive treatment using flexible cystoscopic biopsy and multiparametric magnetic resonance imaging (mpMRI), with subsequent transurethral resection of bladder tumor (TURBT) only if indicated.
**Knowledge Generated**
Over 92% of participants with possible MIBC in Pathway 2 underwent mpMRI after random assignment. After mpMRI, over 22% of participants with possible MIBC in Pathway 2 received correct therapy without the need for TURBT. When comparing median time to correct treatment, patients with MIBC in Pathway 2 (the mpMRI-directed pathway) received correct treatment 45 days sooner than patients with MIBC in Pathway 1 (the TURBT pathway).
**Relevance *(A. Necchi)***
BladderPath is an insightful study that suggests a change in conventional therapeutic paradigm of patients with organ-confined bladder cancer. Reproducibility of MRI protocols and accessibility of advanced imaging modalities will require larger confirmatory studies.*
*Relevance section written by *JCO* Associate Editor Andrea Necchi, MD.

In contrast to many cancers, survival rates from MIBC are not improving,^2^ partly reflecting prolonged pathways. Slow pathways contribute to worse prognosis for MIBC and,^21-23^ in part, reflect the need for TURBT, regardless of health care structure.^22^ In the United Kingdom, patients with MIBC wait an average of 144 days from community referral to radical therapy,^24^ and 48% wait >180 days from diagnosis to cystectomy.^25^ In the United States, patients wait an average of 69 days from TURBT to radical treatment, but with an unknown delay from community referral to TURBT.^21^ In Canada, the wait is up to 56 days to see a urologist before TURBT^26^ and 65 days from TURBT to cystectomy.^27^ A delay of ≥56 days from diagnosis to neoadjuvant chemotherapy is associated with pathologic upstaging,^28^ and delays from diagnosis to radical cystectomy are associated with increased mortality.^29,30^ Conversely, survival advantages are observed in patients undergoing radical cystectomy within 90 days of diagnosis.^31,32^ The picture is worse for women, who are frequently initially misdiagnosed.^26,33^

We hypothesized a better approach is to separate NMIBC and MIBC at diagnosis. We undertook BladderPath^34^ to test whether multiparametric (mp)MRI after flexible cystoscopy and tumor biopsy could discriminate NMIBC and MIBC,^35,36^ and so offer a faster route to definitive treatment by removing the need for TURBT in some cases.

## PATIENTS AND METHODS

### Study Design

BladderPath was an open-label, randomized controlled trial conducted in 17 UK hospitals.^37^ Two stages assessed feasibility and time to correct treatment (TTCT). Patients with suspected BC were recruited from urologic clinics. Informed consent was collected before flexible cystoscopy for urine collection,^38^ and after for random assignment in those with suspected BC. Eligible patients had a suspected diagnosis of new BC, were treatment-naïve, and standard care required TURBT. Alongside flexible cystoscopy, all patients underwent upper tract imaging (ultrasound scanning, CT, or intravenous urography). Excluded patients were unable or unwilling to undergo MRI, those with previous BC, and those previously enrolled. The detailed protocol is available online.^39^

### Random Assignment and Masking

**FIG A1.**
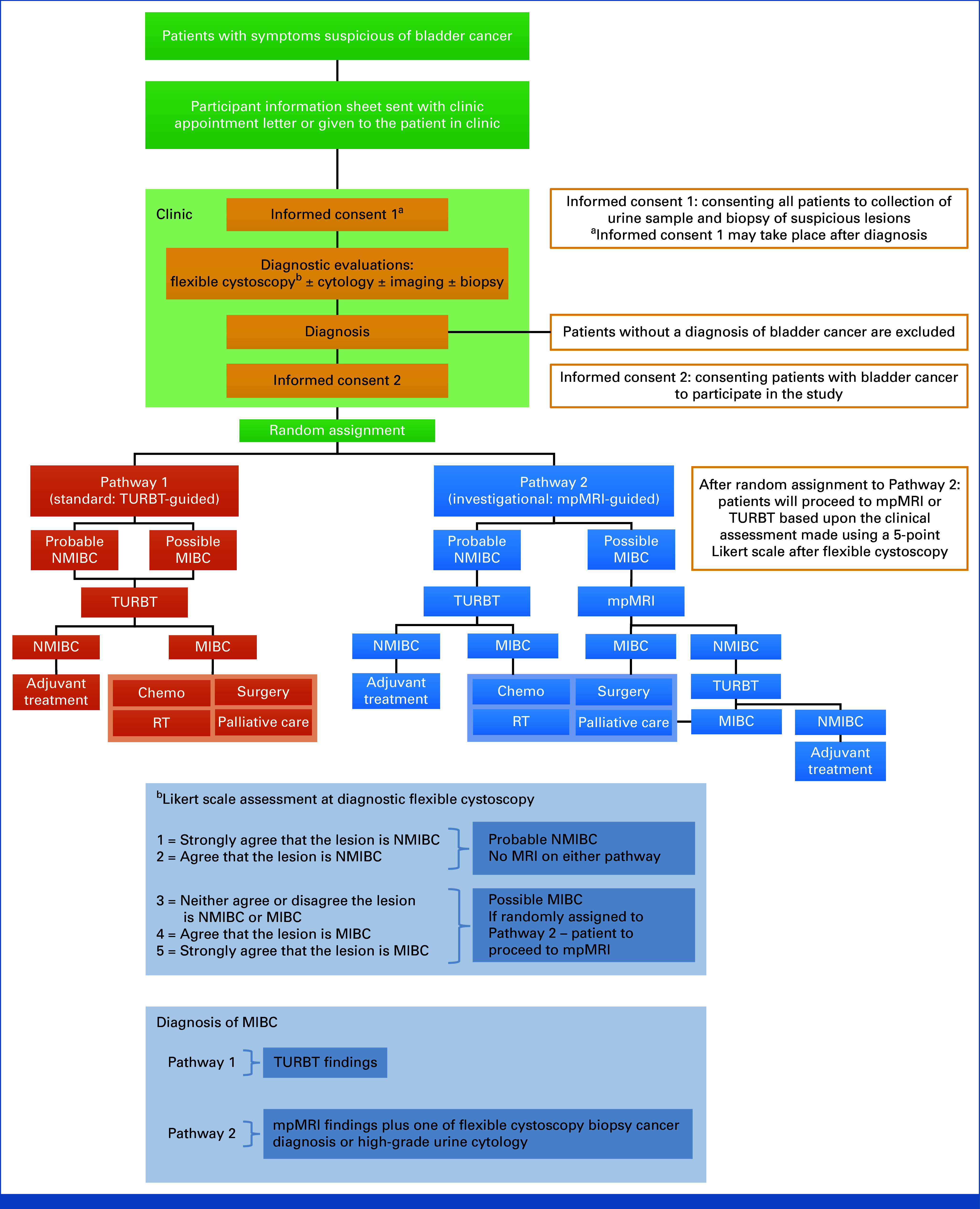
BladderPath study schema. chemo, chemotherapy; MIBC, muscle-invasive bladder cancer; mpMRI, multiparametric magnetic resonance imaging; NMIBC, non–muscle-invasive bladder cancer; RT, radiotherapy; TURBT, transurethral resection of bladder tumor.

Participants were randomly assigned 1:1 to Pathway 1 (TURBT-staged) or Pathway 2 (mpMRI-staged) with minimization factors: sex (male/female), age (<75/≥75 years), and clinician assessment at flexible cystoscopy (probable NMIBC/possible MIBC; Appendix Fig A1, online only, for study schema). Assessment of likelihood of NMIBC or MIBC was based on a 5-point Likert scale: (1) strongly agree or (2) agree that the lesion is NMIBC, or (3) equivocal, or (4) agree or (5) strongly agree that the lesion is MIBC. Likert 1-2 were considered probable NMIBC, and 3-5 were considered possible MIBC. Illustrated posters in cystoscopy clinics facilitated Likert score designation. Additional criteria included high-grade urothelial cells in urine or flexible cystoscopy biopsies, or suspected muscle invasion on diagnostic cross-sectional imaging. Random assignment was unblinded.

### Procedures

Participants randomly assigned to Pathway 1 underwent TURBT as per standard of care (SOC).^6^ Participants randomly assigned to Pathway 2 underwent TURBT if visually assessed as probable NMIBC (Likert 1-2) or mpMRI for possible MIBC (Likert 3-5). For the latter, suspicious lesions were biopsied during outpatient flexible cystoscopy or urine cytology collected. mpMRI imaging was protocol-driven (Appendix 1); sequences and image reporting were VI-RADS–compatible.^36^ After mpMRI, TURBT was permitted at clinicians' discretion to determine histologic variants, for tumor debulking before chemoradiotherapy, diagnostic uncertainty, to assess operability, carcinoma in situ (CIS) assessment, prostatic urethral biopsies for neobladder consideration, restaging after neoadjuvant chemotherapy, or for symptom management. All treatments were determined by local hospital multidisciplinary teams in accordance with national guidelines.^4,6^

### Outcomes

#### Feasibility Stage

The primary outcome was the proportion of participants with possible MIBC randomly assigned to Pathway 2 who correctly followed pathway protocol.^34^ Secondary outcomes included proportion of all randomly assigned participants who correctly followed protocol on each pathway, recruitment and retention rates at each site, and counts of each type of correct treatment.

#### TTCT Stage

The primary outcome was TTCT for participants classified as possible MIBC and confirmed to have MIBC. Secondary outcomes included TTCT for all randomly assigned participants and TTCT for participants with probable NMIBC confirmed as NMIBC. The correct treatment for NMIBC was TURBT. The correct treatment for MIBC was either systemic chemotherapy, radiotherapy, cystectomy, or palliative care. The final MIBC/NMIBC designation was based on cystectomy or TURBT histology, or radiologic findings. All patients had pathologic confirmation of BC.

### Statistical Analysis

For feasibility, the target sample size was 150 participants (c.38 participants with possible MIBC in Pathway 2). If the proportion of participants with possible MIBC randomly assigned to Pathway 2 who correctly followed pathway protocol exceeded 80%, Pathway 2 would be considered feasible in clinical practice.

For the TTCT stage, we assumed a median SOC TTCT of 100 days for participants with MIBC (on the basis of a pretrial audit) and that mpMRI would reduce median TTCT to 70-days, corresponding to a hazard ratio (HR) of 3.6, that is, it would be 3.6× quicker to receive correct treatment for participants with MIBC undergoing mpMRI compared with those undergoing TURBT (assuming hazard of treatment followed a Weibull distribution in both pathways). For 80% power to detect a HR of 3.6, we required 20 participants with MIBC to have received correct treatment. TTCT for participants with MIBC in the two pathways was compared using a Cox regression model adjusted for age and sex, with study center included as a random effect (Appendix Table A1). The same approach applied to secondary outcomes. For censored outcomes, date last seen was used to account for how long a participant awaited treatment.

For feasibility, the population was the participants with possible MIBC randomly assigned to Pathway 2. The populations for the secondary outcomes were the participants allocated to each pathway and those who received each treatment, respectively. For the TTCT stage, time-to-event analyses (an event is receiving a correct treatment) were conducted on an intention-to-treat basis retaining patients in their randomized pathway groups. Statistical analysis was conducted using STATA v17.

Data were supplied confidentially to an independent data monitoring committee (DMC) who advised on whether the accumulated data justified continuation of recruitment. DMC meetings were scheduled at least annually until the study closed to recruitment.

With recruitment affected by COVID-19, it was unfeasible to reach the sample size required for a final clinical stage on the basis of time to progression. A decision to close recruitment after sufficient participants for the first two stages had been recruited was made in discussion with the funder. After opening on May 31, 2018, the trial closed to recruitment on December 31, 2021. The database was locked on September 20, 2022, allowing follow-up for time-to-event outcomes.

### Registration

BladderPath was sponsored by the University of Birmingham, approved by London Bridge Research Ethics Committee (17/LO/1819), and registered as ISRCTN 35296862.

## RESULTS

### Participants

**FIG 1.**
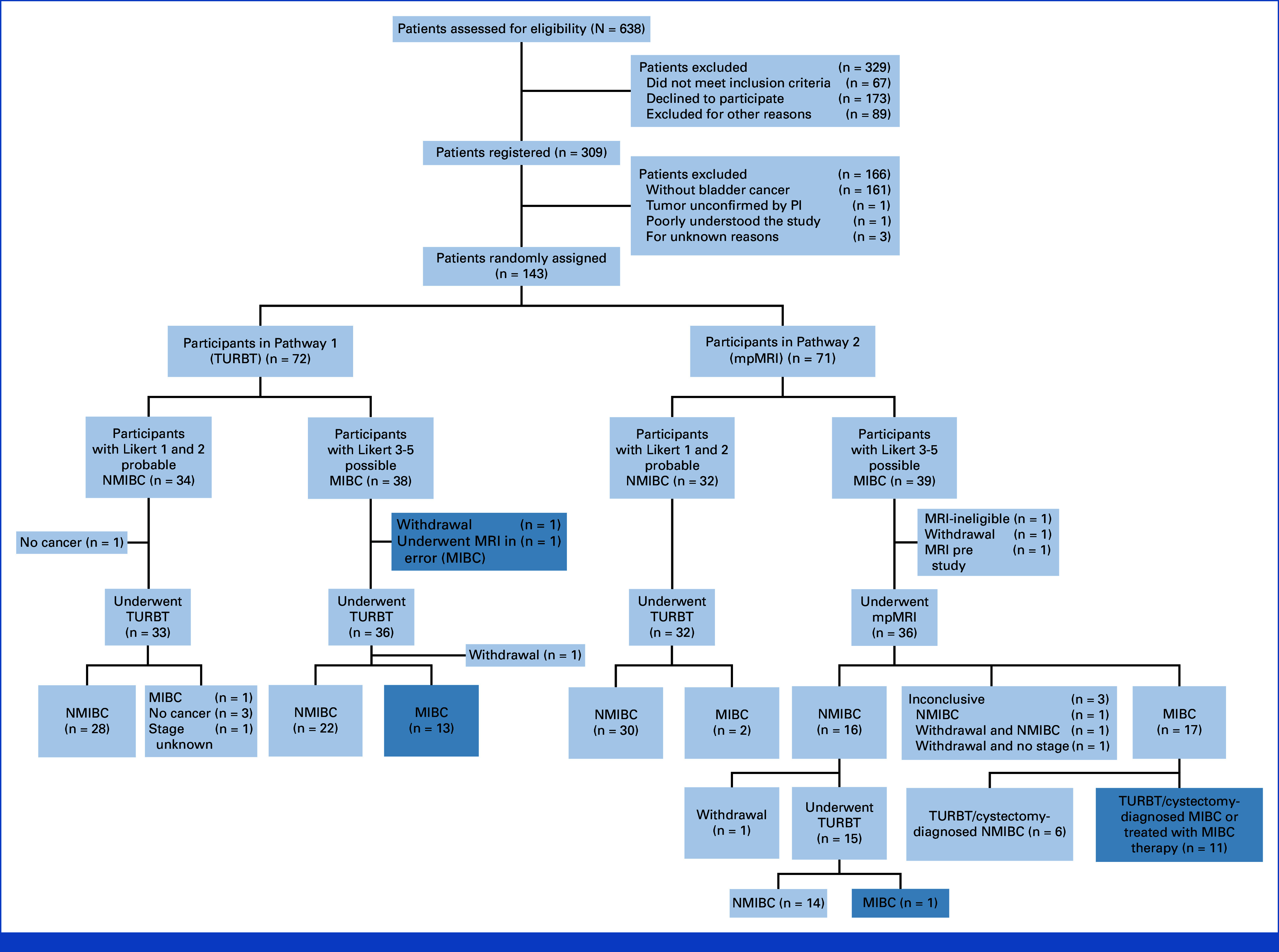
CONSORT diagram. Shaded boxes: Population in primary outcome analysis for time to correct treatment, TTCT (patients with probable MIBC confirmed as MIBC by TURBT or cystectomy, or treated with MIBC therapy, n = 14 in Pathway 1 and n = 12 in Pathway 2). For full NIHR Health Technology Assessment report, please refer to the study by James et al.^37^ MIBC, muscle-invasive bladder cancer; NIHR, National Institute for Health Research; NMIBC, non–muscle-invasive bladder cancer; PI, principal investigator; TTCT, time to correct treatment; TURBT, transurethral resection of bladder tumor.

Between May 31, 2018, and December 31, 2021, 17 hospitals opened to recruitment; 638 patients were screened and 143 participants randomly assigned, 72 to Pathway 1 and 71 to Pathway 2 (Fig 1; Table 1).^37^ Three participants were ineligible after random assignment (one in Pathway 1, two in Pathway 2). Seven participants, including three who did not have cancer, withdrew from the study (three from Pathway 1, four from Pathway 2). Nine protocol deviations were reported for nine participants (five in Pathway 1, four in Pathway 2). Stratification factors were balanced between the pathways, including initial clinical assessment of probable NMIBC/possible MIBC.

**TABLE 1.** Participant Characteristics

Allocation	Pathway 1: TURBT (n = 72), No. (%)	Pathway 2: mpMRI (n = 71), No. (%)	Overall (N = 143), No. (%)
Treating site			
Arrowe Park Hospital	3 (4.2)	2 (2.8)	5 (3.5)
Coventry and Warwickshire Hospital	4 (5.6)	4 (5.6)	8 (5.6)
Derriford Hospital	16 (22.2)	13 (18.3)	29 (20.3)
Glan Clwyd Hospital	3 (4.2)	1 (1.4)	4 (2.8)
Manchester Royal Infirmary	1 (1.4)	0 (0.0)	1 (0.7)
Morriston Hospital	11 (15.3)	15 (21.1)	26 (18.2)
New Cross Hospital	3 (4.2)	1 (1.4)	4 (2.8)
Norfolk and Norwich University Hospital	2 (2.8)	5 (7.0)	7 (4.9)
Northwick Park Hospital	1 (1.4)	0 (0.0)	1 (0.7)
Sheffield Teaching Hospitals	7 (9.7)	17 (23.9)	24 (16.8)
Royal Marsden Hospital London	0 (0.0)	1 (1.4)	1 (0.7)
Royal Oldham Hospital	2 (2.8)	1 (1.4)	3 (2.1)
Royal Stoke University Hospital	1 (1.4)	0 (0.0)	1 (0.7)
St James's University Hospital	3 (4.2)	1 (1.4)	4 (2.8)
The Queen Elizabeth Hospital	15 (20.8)	10 (14.1)	25 (17.5)
Sex			
Male	55 (76.4)	53 (74.6)	108 (75.5)
Female	17 (23.6)	18 (25.4)	35 (24.5)
Age, years			
<75	48 (66.7)	49 (69.0)	97 (67.8)
≥75	24 (33.3)	22 (31.0)	46 (32.2)
Initial clinician assessment			
Probable NMIBC	34 (47.2)	32 (45.1)	66 (46.2)
Possible MIBC	38 (52.8)	39 (54.9)	77 (53.8)
WHO performance status			
0	54 (75.0)	52 (73.2)	106 (74.1)
1	8 (11.1)	10 (14.1)	18 (12.6)
2	3 (4.2)	2 (2.8)	5 (3.5)
3	3 (4.2)	2 (2.8)	5 (3.5)
Unknown	4 (5.6)	5 (7.0)	9 (6.3)
eGFR (value used for eligibility assessment ≥40 mL/min/1.73 m^2^)			
Mean (SD)	74.0 (16.2)	72.7 (16.2)	73.4 (16.1)
Median	79.0	78.0	78.0
IQR	60.5-89.0	60.0-88.0	60.0-88.0
Range	30.0-99.0	39.0-113.0	30.0-113.0
Smoking history			
Nonsmoker	19 (26.4)	25 (35.2)	44 (30.8)
Ex-smoker	40 (55.5)	35 (49.3)	75 (52.4)
Smoker	11 (15.3)	7 (9.9)	18 (12.6)
Unknown	2 (2.7)	4 (5.6)	6 (4.2)
No. of lesions at flexible cystoscopy (where recorded)			
No.	61	63	124
Mean (SD)	1.8 (1.8)	1.7 (1.6)	1.8 (1.7)
Median	1.0	1.0	1.0
IQR	1.0-2.0	1.0-2.0	1.0-2.0
Range	1.0-10.0	1.0-10.0	1.0-10.0
Largest dimension of lesion in cm (where recorded)			
No.	55	59	114
Mean (SD)	2.9 (1.9)	3.2 (1.7)	3.0 (1.8)
Median	2.5	3.0	3.0
IQR	1.5-3.0	2.0-4.0	2.0-4.0
Range	0.2-10.0	0.5-10.0	0.2-10.0
Biopsies at flexible cystoscopy			
No	53 (73.6)	54 (76.1)	107 (74.8)
Yes	16 (22.2)	15 (21.1)	31 (21.7)
Unknown	3 (4.2)	2 (2.8)	5 (3.5)
Description of the most significant lesion at flexible cystoscopy			
Flat and solid	1 (1.4)	0 (0.0)	1 (0.7)
Papillary	32 (44.4)	41 (57.7)	73 (51.0)
Papillary and solid	9 (12.5)	9 (12.7)	18 (12.6)
Solid	22 (30.6)	14 (19.7)	36 (25.2)
Unknown	8 (11.1)	7 (9.9)	15 (10.5)

NOTE. For full NIHR Health Technology Assessment report, please see the study by James et al.^37^

Abbreviations: eGFR, estimated glomerular filtration rate; MIBC, muscle-invasive bladder cancer; mpMRI, multiparametric magnetic resonance imaging; NIHR, National Institute for Health Research; NMIBC, non–muscle-invasive bladder cancer; SD, standard deviation; TURBT, transurethral resection of bladder tumor.

At reporting, 130 (91%) participants had received a correct treatment (Table 2). For the 13 remaining, three did not have BC, three withdrew early (<100 days), one died, two participants with probable NMIBC diagnosed as MIBC after TURBT were awaiting correct treatment, and four participants with probable NMIBC unconfirmed as MIBC/NMIBC were awaiting correct treatment. Of the 143 participants, 132 (92%) had confirmed NMIBC or MIBC, including 50 NMIBCs in Pathway 1, 53 NMIBCs in Pathway 2, 15 MIBCs in Pathway 1, and 14 MIBCs in Pathway 2.

**TABLE 2.** Correct Treatment(s) Received by Pathway for All Participants

Arm	Pathway 1: TURBT (n = 72)	Pathway 2: mpMRI (n = 71)	Overall (N = 143)
Whether or not correct treatment received, No. (%)			
No	9 (12.5)	4 (5.6)	13 (9.1)
Yes	63 (87.5)	67 (94.4)	130 (90.9)
First correct treatment, No. (%)			
Chemotherapy	4 (5.6)	5 (7.0)	9 (6.3)
Cystectomy	2 (2.8)	3 (4.2)	5 (3.5)
Palliative care	4 (5.6)	3 (4.2)	7 (4.9)
Radiotherapy	3 (4.2)	2 (2.8)	5 (3.5)
TURBT	50 (69.4)	54 (76.1)	104 (72.7)
Not known	9 (12.5)	4 (5.6)	13 (9.1)
Time to a correct treatment, days, median (95% CI)	37 (23 to 47)	25 (18 to 35)	31 (22 to 37)
Time to chemotherapy, days, median (95% CI)	66 (42 to NR)	54 (22 to NR)	66 (22 to 81)
Time to cystectomy, days, median (95% CI)	81 (81 to NR)	74 (51 to NR)	81 (51 to NR)
Time to palliative, days, median (95% CI)	125 (76 to NR)	20 (8 to NR)	76 (8 to 138)
Time to radiotherapy, days, median (95% CI)	116 (98 to NR)	69 (69 to NR)	98 (69 to NR)
Time to TURBT, days, median (95% CI)	18 (12 to 29)	20 (17 to 29)	20 (16 to 26)

NOTE. For full NIHR Health Technology Assessment report, please see the study by James et al.^37^

Abbreviations: mpMRI, multiparametric magnetic resonance imaging; NIHR, National Institute for Health Research; NR, not reached; TURBT, transurethral resection of bladder tumor.

### Feasibility Stage

**FIG 2.**
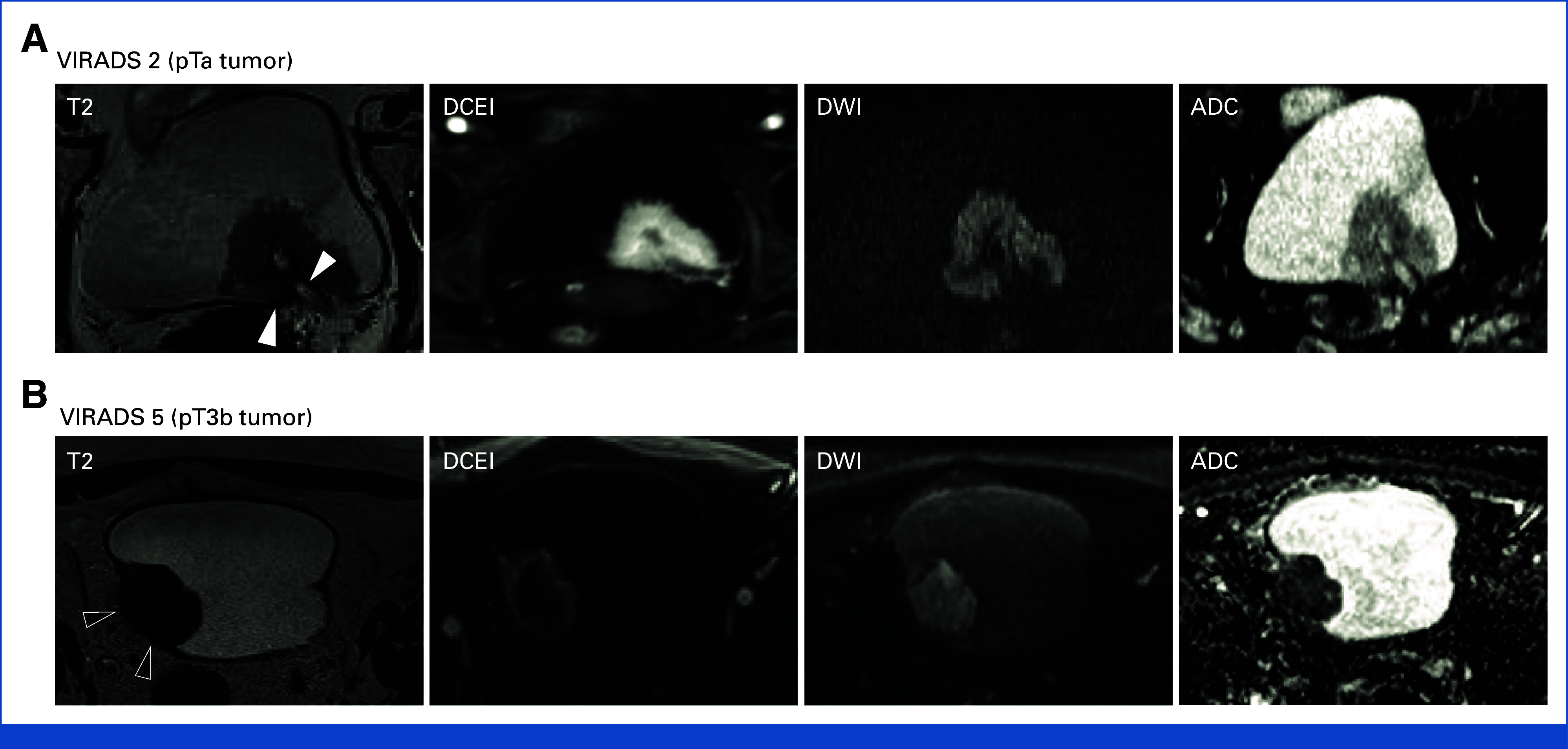
Examples of mpMRI images from recruited participants for (A) noninvasive (pTa) and (B) invasive (pT3b) bladder cancers initially thought to be possible MIBC (Likert 3-5) at flexible cystoscopy. White arrows indicate intact muscularis propria. Black arrows indicate loss of normal muscularis propria because of invasive tumor. For the full NIHR Health Technology Assessment report, please refer to the study by James et al.^37^ ADC, apparent diffusion coefficient; DCEI, dynamic contrast enhancement image; DWI, diffusion-weighted imaging; MIBC, muscle-invasive bladder cancer; mpMRI, multiparametric magnetic resonance imaging; NIHR, National Institute for Health Research; T2, MRI T2 weighted; VIRADS, Vesical Imaging-Reporting and Data System.

As reported,^34^ 36 of 39 (92%, 95% CI, 79 to 98) participants with possible MIBC in Pathway 2 underwent mpMRI after random assignment. Of the three who did not, one participant had metal in their eye, one participant withdrew (29 days after random assignment), and one underwent pretrial MRI outside the study. The overall proportion of participants correctly following their respective protocol pathway was 96% (95% CI, 88 to 99) in each pathway (no statistical difference between pathways). Of the 36 participants who underwent mpMRI, 33 were fully compliant with the VI-RADS protocol (92%) and 34 (94%) demonstrated optimal bladder filling. Example images are shown in Figure 2.

### Time to Correct Treatment Stage

**FIG 3.**
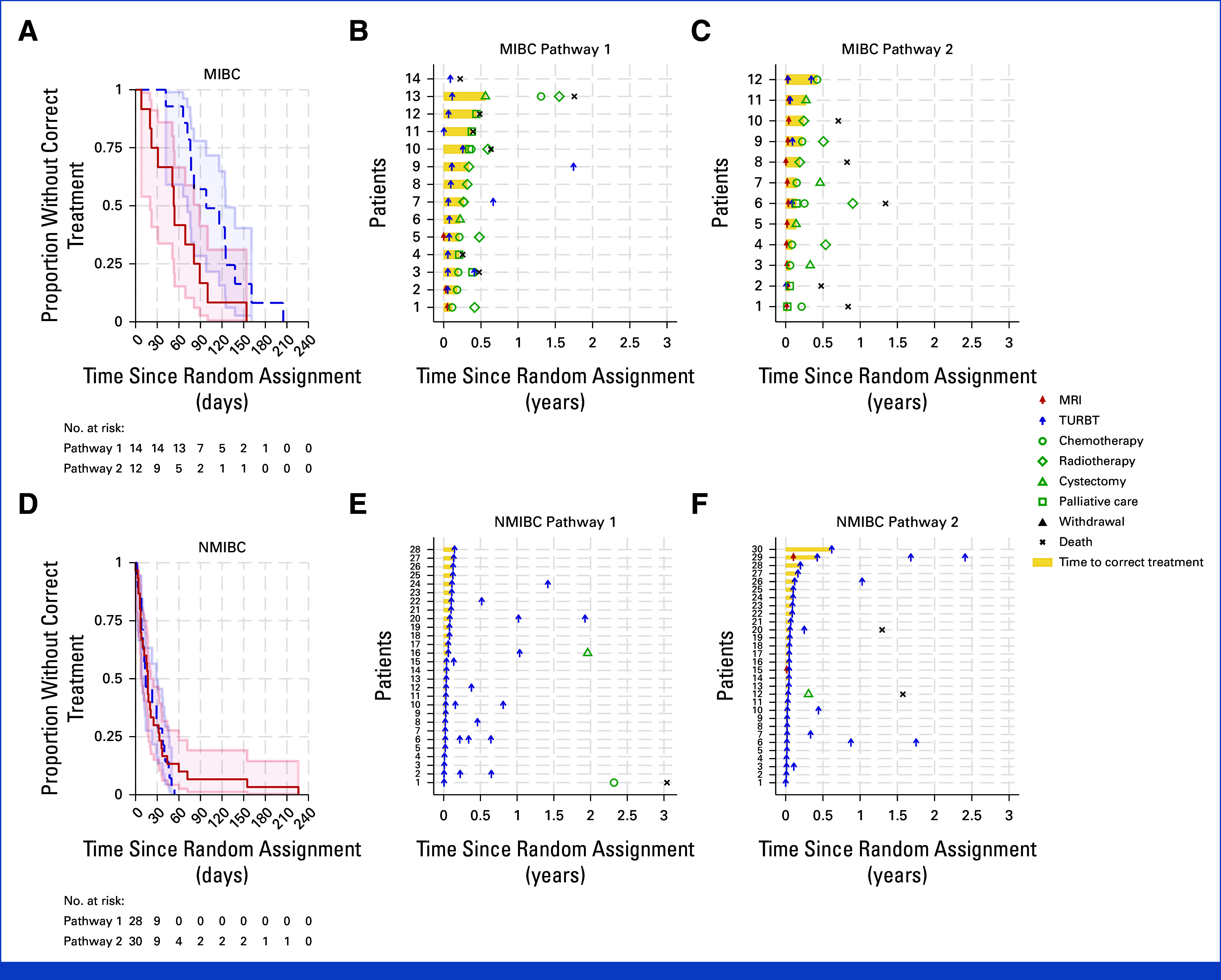
(A) Kaplan-Meier curves of TTCT by pathway for participants with possible MIBC who were confirmed MIBC and received a correct treatment; (B) swimmer plot for Pathway 1 participants with possible MIBC; (C) swimmer plot for Pathway 2 participants with possible MIBC; (D) Kaplan-Meier curves of TTCT by pathway for participants with probable NMIBC who were confirmed NMIBC and received a correct treatment; (E) swimmer plot for Pathway 1 participants with probable NMIBC; and (F) swimmer plot for Pathway 2 participants with probable NMIBC. For the Kaplan-Meier curves, blue dashed lines represent Pathway 1 (TURBT pathway) and red lines represent Pathway 2 (mpMRI-guided pathway). For the swimmer plots, the yellow bars illustrate TTCT. For full NIHR Health Technology Assessment report, please refer to the study by James et al.^37^ MIBC, muscle-invasive bladder cancer; mpMRI, multiparametric magnetic resonance imaging; MRI, magnetic resonance imaging; NIHR, National Institute for Health Research; NMIBC, non–muscle-invasive bladder cancer; TTCT, time to correct treatment; TURBT, transurethral resection of bladder tumor.

Our primary outcome was TTCT for participants with possible MIBC. Of 26 participants assessed as possible MIBC and confirmed as MIBC (14 in Pathway 1, 12 in Pathway 2), 25 received a correct treatment (the remaining participant died 81 days after random assignment before correct treatment). Median TTCT for all 26 participants with MIBC was 77 days (95% CI, 54 to 100). When compared, Pathway 2 appeared significantly quicker (log-rank *P* = .02, Fig 3): median TTCT for Pathway 1 (n =14) was 98 days (95% CI, 72 to 125) and for Pathway 2 (n = 12) was 53 days (95% CI, 20 to 89). A Cox model adjusting for stratification factors of sex and age, with study center included as a random effect, demonstrated an HR in favor of Pathway 2 versus Pathway 1 of 2.9 (95% CI, 1.0 to 8.1).

**FIG A2.**
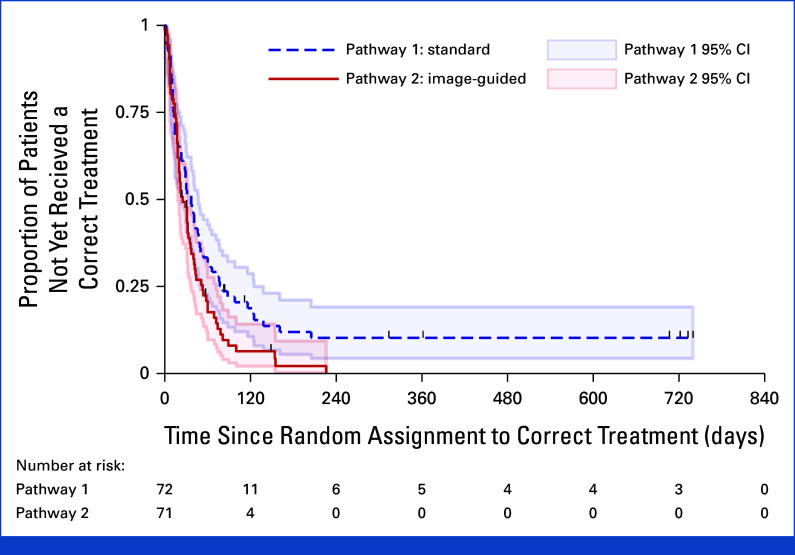
Kaplan-Meier curves of time to correct treatment by pathway for all randomly assigned participants.

Our secondary outcomes included TTCT for participants with probable NMIBC and for all recruited. There were 58 participants designated probable NMIBC, confirmed histologically as NMIBC (28 in Pathway 1, 30 in Pathway 2), and all received TURBT as correct treatment. The median TTCT for these 58 participants was 16 days (95% CI, 11 to 23). No differences (log-rank *P* = .67, Fig 3) were seen between pathways: median TTCT for Pathway 1 (n = 28) was 14 days (95% CI, 10 to 29) and 17 days (95% CI, 8 to 25) for Pathway 2 (n = 30). A Cox model adjusting for the stratification factors of sex and age demonstrated an HR for Pathway 2 versus Pathway 1 of 0.8 (95% CI, 0.5 to 1.5). When all randomly assigned participants were analyzed, 131 of 143 (91.6%) received a correct treatment (those who had not yet received a correct treatment were censored at their date last seen and included in the time-to-treatment analysis). Median TTCT for all 143 participants was 31 days (95% CI, 22 to 37) and faster for Pathway 2 (log-rank *P* = .03. Appendix Fig A2): median TTCT for Pathway 1 (n = 72) was 37 days (95% CI, 23 to 47) and 25-days (95% CI, 18 to 35) for Pathway 2 (n = 71). A Cox model adjusting for the stratification factors of sex and age showed an HR for Pathway 2 versus Pathway 1 of 1.4 (95% CI, 0.9 to 2.0), demonstrating no evidence that shorter TTCT for participants with MIBC was achieved to the detriment of TTCT for participants with NMIBC.

### TURBT After mpMRI-Diagnosed MIBC in Pathway 2

Eight participants underwent TURBT after mpMRI-diagnosed MIBC (8 of 17, 47%). Reasons for this were (including multiple for some patients) debulking before radical radiotherapy (4), concern regarding histologic variants (5) or presence of CIS (1), and uncertainty over mpMRI findings (3).

### Histological Outcomes for Cystectomy Patients

A total of 20 cystectomies were undertaken (Appendix Table A2): eight in Pathway 1 (one no tumor [pT0], five NMIBCs, two MIBCs [pT3b and pT4a]) and 12 in Pathway 2 (one no tumor [pT0], six NMIBCs, four MIBCs [two pT2, pT3a, and pT3b], and one unknown stage). The only case of nodal metastases was in Pathway 1. There was no statistical difference in the number of cystectomies undertaken for NMIBC between the two pathways (Fisher exact test *P* = .4). All patients diagnosed NMIBC at cystectomy had undergone previous TURBT; no patient underwent cystectomy because of incorrect mpMRI staging (Table 3).

**TABLE 3.** Treatment Pathway for Participants Who Did Not Have MIBCs Confirmed by Cystectomy (each row represents a participant)

Pathway	Initial Assessment	mpMRI Diagnosis	First TURBT Stage	Second TURBT Stage	Cystectomy Stage
1	Possible MIBC		pT1		pT1
1	Probable NMIBC		pT1		pT0
1	Possible MIBC		pT1		pTa
1	Possible MIBC		pTa		pTa
1	Possible MIBC		pT1		pTis
1	Possible MIBC		pT1		pTis
2	Possible MIBC	NMIBC	pT1	pTa	pTa
2	Possible MIBC		pT1		pTa
2	Possible MIBC	NMIBC	pT1		pT0
2	Possible MIBC	MIBC	pTa	pTa	pTis
2	Possible MIBC	MIBC	pTa		pT1
2	Possible MIBC	Inconclusive	pT1		pT1
2	Possible MIBC	MIBC	T2		pTis

NOTE. For full NIHR Health Technology Assessment report, please see the study by James et al.^37^ Pathway 1 = TURBT pathway, Pathway 2 = mpMRI pathway.

Abbreviations: MIBC, muscle-invasive bladder cancer; mpMRI, multiparametric magnetic resonance imaging; NIHR, National Institute for Health Research; NMIBC, non–muscle-invasive bladder cancer; TURBT, transurethral resection of bladder tumor.

### Adverse Events

No serious adverse events were reported.

### Clinical Outcomes

Median follow-up duration was 23.7 months (95% CI, 23.7 to 24.0) overall: 23.7 months for Pathway 1 (n = 72; 95% CI, 23.7 to 24.0) and 24.0 months for Pathway 2 (n = 71; 95% CI, 23.7 to 24.1). At reporting, there were 70 recurrence, progression, or new primary events from 47 participants (Appendix Table A3). Metastatic disease was seen in 13 participants, including 10 (26%) from Pathway 1 and 3 (10%) from Pathway 2. Death occurred in 20 participants including disease-related mortality for 7 (70%) participants from Pathway 1 and 3 (30%) from Pathway 2 (Appendix Table A4).

## DISCUSSION

We have demonstrated it is feasible and safe to omit TURBT after mpMRI for a proportion of patients visually assessed as MIBC at flexible cystoscopy. Importantly, mpMRI staging expedited definitive treatment (by over 6 weeks) for patients with MIBC. When all participants were analyzed, the faster TTCT for MIBC was achieved with no detriment to participants with NMIBC. This is impressive, given some participants with mpMRI-staged MIBC still underwent TURBT for histologic clarification.

Delays to definitive treatment for MIBC are ubiquitous and deleterious.^21-32,40^ TURBT contributes to these delays, leading to incorrect treatments or tumor spread.^11,14,15,41^ Multiple studies report mpMRI has high diagnostic accuracy for MIBC, with sensitivity up to 89% and specificity up to 86%.^35,36,42^ With a 6%-8% increase in the risk of death for every 28-day delay to definitive treatment,^23^ one can assume administering treatment >45 days earlier than the current SOC is only beneficial.

TURBT is considered necessary for resecting the luminal BC component for histologic categorization, symptom relief, or reducing tumor volume before bladder-sparing radical treatment.^43^ However, the literature shows that TURBT and cystectomy histology are often discordant,^44^ understaging by TURBT is common,^16,17,41,45^ and there are few data to suggest tumor debulking is a necessary therapeutic (as opposed to prognostic) component of radiotherapy.^46^ Furthermore, flexible cystoscopic biopsies yield enough material for both histopathologic diagnosis of cancer and molecular subtyping.^47,48^

There are limitations to our work. First, COVID-19 interrupted recruitment and so we did not enroll sufficient participants for survival outcomes. Nevertheless, a comparable practice change in prostate cancer did not require survival-based studies,^49^ although it was intriguing that the number of metastatic and disease-related mortality events appeared to favor Pathway 2. Second, the pathologic stage in participants who underwent systemic chemotherapy, radiotherapy, or palliation for mpMRI-diagnosed MIBC was unknown, and it is impossible to know whether these were correct treatments. Given the limitations of TURBT, there is no perfect ground truth on either side of the random assignment. The mpMRI-based Vesical Imaging-Reporting and Data System (VI-RADS^36^) was introduced during the conduct of our study. VI-RADS provides a separate, peer-reviewed classification system for implementation of mpMRI in this setting (as for prostate cancer diagnosis) and so rollout of these pathways should be possible. Notwithstanding, 6 of 17 patients reported as MIBC by mpMRI in Pathway 2 were subsequently diagnosed as NMIBC, suggesting that there is a learning curve to overcome before optimal MIBC diagnosis; with experience, we expect MIBC false-positive rates to fall, analogous to prostate cancer.^50^ Consequently, a proportion of patients in Pathway 2 underwent TURBT after mpMRI, including four for histologic clarification or three for lack of confidence that the MRI shows MIBC; regardless, within our trial, these participants still had shorter TTCT than with SOC. Notably, a proportion of patients (eg, those with metastases) avoided TURBT completely, sparing resources and reducing their morbidity.

An important component of our pathway is triage of patients into probable NMIBC or MIBC at the time of flexible cystoscopy. As previously demonstrated,^51^ we have shown this is possible and accurate (89% of lesions visually classified as NMIBC were confirmed as NMIBC). For the remainder, mpMRI provided rapid accurate triage with an approximately equal split between NMIBC and MIBC. Although analogous to prostate cancer diagnosis,^49^ with initial cystoscopic triage and lower numbers of BC cases, impact upon departmental MRI workload is modest.

Detailed health-economic analysis is beyond current scope. However, with TURBT costing c.10× more than mpMRI, omitting TURBT is likely cost-saving if >1/10 mpMRIs lead to this outcome. In BladderPath, 8 of 36 patients (22%) underwent definitive therapy or palliative care that did not require TURBT; hence, overall, the pathway should be cost-saving, while liberating capacity for NMIBC TURBTs.

Experience with introduction of mpMRI into the prostate cancer pathway has been that, after initial skepticism, there is widespread acceptance of the paradigm that more accurate imaging improves clinical decision making.^52^ Notably, this adoption has occurred in the absence of randomized survival-based studies. For the BC pathway, often managed by the same teams, introduction of mpMRI is likely to be more straightforward—our experience with BladderPath suggests that clinicians rapidly become comfortable with using mpMRI to guide downstream decisions.

In conclusion, incorporating mpMRI ahead of TURBT into the standard pathway was beneficial for all patients with suspected MIBC. TURBT could be avoided in a proportion of these patients. This approach can improve decision making and accelerate time to treatment.

## Data Availability

Participant data and the associated supporting documentation will be available within 6 months after the publication of the study results in a peer-reviewed journal. Details of our data request process is available on the CRCTU website. Only scientifically sound proposals from appropriately qualified research groups will be considered for data sharing. The decision to release data will be made by the Trial Management Group who will consider the scientific validity of the request, the qualifications and resources of the research group, the views of the CRCTU Director's Committee and the Trial Steering Committee, consent arrangements, the practicality of anonymizing the requested data and contractual obligations. A data sharing agreement will cover the terms and conditions of the release of trial data and will include publication requirements, authorship and acknowledgments, and obligations for the responsible use of data. An anonymized encrypted data set will be transferred directly using a secure method and in accordance with the University of Birmingham's IT guidance on encryption of data sets.

## APPENDIX 1. MAGNETIC RESONANCE IMAGING BLADDERPATH PROTOCOL

### Magnetic Resonance Equipment

Magnetic resonance imaging (MRI) 1.5T with multichannel phased array external surface coil and the patients lying supine.

### Image Acquisition

Optimal bladder distension is achieved by instructing the patient to void no later than 2 hours before imaging. Lack of bladder distension will limit the detection of small tumors secondary to detrusor muscle thickening. Artifact from bowel peristalsis can be minimized by using anterior saturation bands.

T_1_ weighted spin echo (T_1_W-SE), T_2_ weighted turbo spin echo (T_2_W-TSE), high resolution T_2_W-TSE, diffusion-weighted imaging (DWI), and dynamic contrast enhanced (DCE) MRI are key components. Images should include the whole bladder, pelvic nodes, and proximal urethra. In males, prostate, and in females, the uterus, ovaries, fallopian tubes, and vagina should also be included. T_1_W-SE axial is used to identify hemorrhage and clot in the bladder and bone metastasis.

If possible, administration of Buscopan is recommended and should be performed according to the local policy.

In general, small deviations from the scanning parameters are allowed (ie, changing bandwidth, field of view (FOV), or acquisition matrix), especially when planning scans perpendicular to the tumor base, as these might require adjusting FOV and matrix to avoid wrapping. It is suggested in these situations to keep the total voxel dimensions as close as possible to suggested values.

Phase and slice oversampling values should also be treated as guidance because of their high dependence on the patient's size and the orientation of acquired images.

#### T2 Weighted Imaging

High-resolution T_2_W images without fat suppression were obtained with fast spin echo or turbo spin echo sequences, in three orthogonal planes with one plane perpendicular to the tumor base, a small FOV, and a large matrix. A thickness of 3-4 mm is recommended to maximize spatial resolution while maintaining signal to noise ratio.

#### DWI

DWI images should be in the orientation perpendicular to the tumor base. A high b value is needed (800-1,000) to visualize the bladder cancer. If your center currently acquires DWI with b values >1,000, we recommend to do that using a separate acquisition sequence.

### Dynamic Contrast-Enhanced Imaging

3D acquisition with fat suppression (eg, VIBE, LAVA, THRIVE) is ideal because of higher spatial resolution. Precontrast and postcontrast images are obtained in transverse plane with isotropic spatial resolution. Reconstruction of images in the plane perpendicular to the tumor base should be performed on the scanner. Gadolinium-based contrast agent is administered using a power injector system at a dose of 0.1 mmol/kg of body weight at the rate of 2.0 ml/s. Acquire the first DCE (single measurement) sequence as a precontrast reference. Injection should begin with the multidynamic DCE acquisition at a temporal resolution <20 seconds to depict the early enhancement of the inner layer followed by tumor enhancement. The bladder tumor, mucosa, and submucosa enhance early (approximately 20 seconds after contrast injection), but the muscle layer enhances late (1 minute). If the scanner cannot perform under these parameters, it is important to keep the acquisition time no longer than 20 seconds; lowering spatial resolution is acceptable; however, keeping isotropic voxel size is highly advisable (Appendix Table A5).

## APPENDIX 2. BLADDERPATH COLLABORATIVE GROUP

University of Birmingham, UK: Douglas Ward, Naheema Gordon, Sarah Pirrie, Sophia Magwaro, Ann Pope, Ana Hughes, Hiede Doyle.

The Royal Oldham Hospital: Jacob Cherian, Lyndsay Scarratt, Dawn Johnstone, Denise Hadfield, Amy Slack.

Betsi Cadwaladr University Health Board: Kingsley Ekwueme, Amanda Jones, Bethan Wyn Owen.

Royal Stoke Hospital, University Hospitals of North Midlands: Lyndon Gommersall, Mark Kitchen, Michelle Davies, Vinodh Murali.

Northwick Park Hospital, London North West University Healthcare NHS Trust: Giles Hellawell, Prasanna Gurung.

Derriford Hospital, University Hospitals Plymouth NHS Trust: Paul Hunter-Campbell, Lyn Cogley.

Morriston Hospital, Swansea Bay University Health Board: Gokul Kanda Swamy, Ellen Turrell, Maria Johnstone.

Sheffield Teaching Hospitals NHS Trust: James WF Catto, Steven Kennish, Aidan P Noon, Marcus GK Cumberbatch, Derek J Rosario, Louise Goodwin, Katharine Behennah, Ferekh Salim, Syed A Hussain.

St James' University Hospital, Leeds Teaching Hospitals NHS Trust: Sanjeev Kotwal, Christopher Main.

Norfolk and Norwich University Hospitals NHS Foundation Trust: Vivekanandan Kumar, Mark Harmer.

New Cross Hospital, The Royal Wolverhampton NHS Trust: David Mak, Will Taylor, Baljinder Rai.

Manchester University Hospitals NHS Foundation Trust: Amar Mohee, Hosam A Noweir, Houda Chea.

Arrowe Park Hospital, Wirral University Teaching Hospital NHS Foundation Trust: Thiagarajan Nambirajan, Nina Patrick, Vandan Arora, Ya-Wen Jessica Huang.

University College London Hospitals NHS Foundation Trust: Prashant Patel.

University Hospitals Birmingham NHS Foundation Trust: Amar Manandhar, Rosie Henvey.

University Hospitals Coventry and Warwickshire NHS trust: Kieran P Jefferson, Davina Hewitt, Anthony Blacker, Donald MacDonald, James Harding, Yakhub Khan, Altan Omer, Andrew Chan, Diokno Michael.

The Royal Marsden NHS Foundation Trust, London: Nicholas D James, Bernard Sui, Josh Shur, Vincent Khoo.

**TABLE A1.** Stratification Factors for Patients With Confirmed Muscle-Invasive Bladder Cancers Included in Time to Correct Treatment Analysis for Patients Designated as Possible Muscle-Invasive Bladder Cancer

Allocation	Pathway 1: TURBT (n = 72), No. (%)	Pathway 2: mpMRI (n = 71), No. (%)	Overall (N = 143), No. (%)
Treating site			
Derriford Hospital	1 (7.1)	1 (8.3)	2 (7.7)
Morriston Hospital	2 (14.3)	0 (0.0)	2 (7.7)
New Cross Hospital	1 (7.1)	1 (7.1)	2 (7.7)
Norfolk and Norwich University Hospital	1 (7.1)	3 (25.0)	4 (15.4)
Northwick Park Hospital	1 (7.1)	0 (0.0)	1 (3.9)
Sheffield Teaching Hospitals	0 (0.0)	6 (50.0)	6 (23.1)
Royal Stoke University Hospital	1 (7.1)	0 (0.0)	1 (3.9)
St James's University Hospital	1 (7.1)	0 (0.0)	1 (3.9)
The Queen Elizabeth Hospital	6 (42.9)	1 (8.3)	7 (26.9)
Sex			
Male	11 (78.6)	8 (67.7)	19 (73.1)
Female	3 (21.4)	4 (33.3)	7 (26.9)
Age, years			
<75	10 (71.4)	8 (66.7)	18 (69.2)
≥75	4 (28.6)	4 (33.3)	8 (30.8)

Abbreviations: mpMRI, multiparametric magnetic resonance imaging; TURBT, transurethral resection of bladder tumor.

**TABLE A2.** Cystectomy Histology for All Patients

Pathway	Pathway 1: TURBT (n = 8), No. (%)	Pathway 2: mpMRI (n = 12), No. (%)	Overall (N = 20), No. (%)
Histologic composition			
Adenocarcinomatous elements	0 (0.0)	2 (16.7)	2 (10.0)
None	0 (0.0)	1 (8.3)	1 (5.0)
Squamous elements, other	0 (0.0)	1 (8.3)	1 (5.0)
Transitional cell carcinoma	4 (50.0)	6 (50.0)	10 (50.0)
Transitional cell carcinoma, adenocarcinomatous elements	0 (0.0)	1 (8.3)	1 (5.0)
Transitional cell carcinoma, other	2 (25.0)	0 (0.0)	2 (10.0)
Transitional cell carcinoma, squamous elements	2 (25.0)	1 (8.3)	3 (15.0)
Detrusor muscle (tumor base)			
No	3 (60.0)	5 (50.0)	8 (53.3)
Yes	2 (40.0)	5 (50.0)	7 (46.7)
Not known	3	2	5
Tumor present in muscle			
Yes	2 (100.0)	5 (100.0)	7 (100.0)
Not known	6	7	13
Random bladder biopsy			
No	6 (100.0)	11 (100.0)	17 (100.0)
Not known	2	1	3
Cytology			
No	6 (100.0)	11 (100.0)	17 (100.0)
Not known	2	1	3
Other sampling			
APER	0 (0.0)	1 (8.3)	1 (5.0)
NA	8 (100.0)	10 (83.3)	18 (90.0)
Uterus, cervix, adnexa	0 (0.0)	1 (8.3)	1 (5.0)
Bladder carcinoma			
No	0 (0.0)	2 (16.7)	2 (10.0)
Yes	8 (100.0)	10 (83.3)	18 (90.0)
Grade (WHO 1973)			
1	0 (0.0)	0 (0.0)	0 (0.0)
2	0 (0.0)	1 (8.3)	1 (5.0)
3	5 (62.5)	7 (58.3)	12 (60.0)
Unable to determine	2 (25.0)	1 (8.3)	3 (15.0)
Not known	1 (12.5)	3 (25.0)	4 (20.0)
Grade (WHO 2004)			
High	4 (50.0)	8 (66.7)	12 (60.0)
Low	0 (0.0)	1 (8.3)	1 (5.0)
Not known	4 (50.0)	3 (25.0)	7 (35.0)
pT stage			
pT0	1 (12.5)	1 (8.3)	2 (10.0)
pTa	0 (0.0)	2 (16.7)	2 (10.0)
pTis	1 (12.5)	1 (8.3)	2 (10.0)
pT1	1 (12.5)	0 (0.0)	1 (5.0)
pT1a (pCIS)	3 (37.5)	1 (8.3)	4 (20.0)
pT1b (pCIS)	0 (0.0)	2 (16.7)	2 (10.0)
T2	0 (0.0)	2 (16.7)	2 (10.0)
pT3a	0 (0.0)	1 (8.3)	1 (5.0)
pT3b	1 (12.5)	1 (8.3)	2 (10.0)
pT4a	1 (12.5)	0 (0.0)	1 (5.0)
Not known	0 (0.0)	1 (8.3)	1 (5.0)
pN stage			
N0	6 (75.0)	10 (83.3)	16 (80.0)
N1	0 (0.0)	0 (0.0)	0 (0.0)
N2	1 (12.5)	0 (0.0)	1 (5.0)
Not known	1 (12.5)	2 (16.7)	3 (15.0)
Margins/status			
Positive	0 (0.0)	1 (8.3)	1 (5.0)
Negative	7 (87.5)	7 (58.3)	14 (70.0)
Unknown	0 (0.0)	3 (25.0)	3 (15.0)
Not known	1 (12.5)	1 (8.3)	2 (10.0)
Concomitant flat in situ carcinoma			
No	2 (25.0)	5 (41.6)	7 (35.0)
Yes	6 (75.0)	5 (41.6)	11 (55.0)
Not known	0 (0.0)	2 (16.7)	2 (10.0)

Abbreviations: APER, abdominoperineal resection; mpMRI, multiparametric magnetic resonance imaging; TURBT, transurethral resection of bladder tumor.

**TABLE A3.** Recurrence, Metastatic Disease, and New Primary Cancers

Pathway	Pathway 1: TURBT (n = 39), No. (%)	Pathway 2: mpMRI (n = 31), No. (%)	Overall (N = 70), No. (%)
Locoregional disease			
No	7 (17.9)	4 (12.9)	11 (15.7)
Yes	31 (79.5)	26 (83.9)	57 (81.4)
Not known	1 (2.6)	1 (3.2)	2 (2.9)
Upper tract urothelial cancer or urethra			
NA	7 (17.9)	4 (12.9)	11 (15.7)
No	26 (66.7)	23 (74.2)	49 (70.0)
Yes	4 (10.3)	2 (6.5)	6 (8.6)
Not known	2 (5.1)	2 (6.5)	4 (5.7)
Was the recurrence in the same location as the primary tumor			
NA	7 (17.9)	4 (12.9)	11 (15.7)
No	7 (17.9)	9 (29.0)	16 (22.9)
Yes	21 (53.8)	14 (45.2)	35 (50.0)
Not known	4 (10.3)	4 (12.9)	8 (11.4)
Metastatic disease			
No	27 (69.2)	25 (80.6)	52 (74.3)
Yes	10 (25.6)	3 (9.7)	13 (18.6)
Not known	2 (5.1)	3 (9.7)	5 (7.1)
Metastatic site			
Brain	1 (2.6)	0 (0.0)	1 (1.4)
Liver	1 (2.6)	0 (0.0)	1 (1.4)
Lung	3 (7.7)	2 (6.5)	5 (7.1)
Lung, bone	1 (2.6)	0 (0.0)	1 (1.4)
Lung, pelvic nodal, metastatic nodal	1 (2.6)	0 (0.0)	1 (1.4)
NA	27 (69.2)	25 (80.6)	52 (74.3)
Not known	2 (5.1)	3 (9.7)	5 (7.1)
Other	1 (2.6)	1 (3.2)	2 (2.9)
Pelvic nodal	1 (2.6)	0 (0.0)	1 (1.4)
Pelvic nodal, metastatic nodal	1 (2.6)	0 (0.0)	1 (1.4)
New primary tumor			
No	37 (94.9)	25 (80.6)	62 (88.6)
Yes	1 (2.6)	4 (12.9)	5 (7.1)
Not known	1 (2.6)	2 (6.5)	3 (4.3)
Site of new primary tumor			
NA	37 (94.9)	25 (80.6)	62 (88.6)
Not known	1 (2.6)	5 (16.1)	6 (8.6)
Other	0 (0.0)	1 (3.2)	1 (1.4)
Renal	1 (2.6)	0 (0.0)	1 (1.4)

Abbreviations: mpMRI, multiparametric magnetic resonance imaging; TURBT, transurethral resection of bladder tumor.

**TABLE A4.** Summary of Causes of Death

Pathway	Pathway 1: TURBT (n = 10), No. (%)	Pathway 2: mpMRI (n = 10), No. (%)	Overall (N = 20), No. (%)
Cause of death			
Disease-related	7 (70.0)	3 (30.0)	10 (50.0)
Not known	1 (10.0)	3 (30.0)	4 (20.0)
Other cancer—metastatic prostate cancer + metastatic oropharyngeal cancer	1 (10.0)	0 (0.0)	1 (5.0)
Other cancer—leukemia	0 (0.0)	1 (10.0)	1 (5.0)
Other noncancer—COVID-19	0 (0.0)	1 (10.0)	1 (5.0)
Other noncancer—COVID-19 pneumonitis	1 (10.0)	0 (0.0)	1 (5.0)
Other noncancer—multiorgan failure	0 (0.0)	1 (10.0)	1 (5.0)
Other noncancer—pulmonary thromboembolism	0 (0.0)	1 (10.0)	1 (5.0)

Abbreviations: mpMRI, multiparametric magnetic resonance imaging; TURBT, transurethral resection of bladder tumor.

**TABLE A5.** Parameter Settings at 1.5 T

Setting	T_1_W-SE	T_2_W-TSE	HR-T_2_W-TSE^a^	DWI	DCE MRI-VIBE^b^
TR, ms	760	5,000	4,750	4,500	5.47 (shortest)
TE, ms	11	76	105	88	2.39 (shortest)
Flip angle, degree	180	90	150	90	10
FOV, cm	400 × 400	230 × 230	160 × 160	270 × 270	320 × 240
Matrix	204 × 256	256 × 256	256 × 256	109 × 128	160 × 160
Voxel size, mm	2.0 × 1.6	0.9 × 0.9	0.6 × 0.6	2.5 × 2.1	2.0 × 2.0
Phase oversampling	30%^c^	50%^c^	100%^c^	30%^c^	0%^c^
Slice oversampling	**—**	**—**	**—**	**—**	16.7%^c^
Slice thickness, mm	5	4	3-4	4	2.0
No. of slices	25^c^	27^c^	23^c^	23^c^	48^c^
Slice gap, mm	1.25 (25%)	0.4 (10%)	0.3-0.4 (10%)	0.4 (10%)	0
Orientation	Transverse	Sagittal	Perp tumor	Perp tumor	Transverse
No. of averages	3	2	3	10	1
Bandwidth, Hz/Px	186	130	130	1,698	290
Acquisition time	2:36	2:37	4:37	5:30	0:20
Acceleration fact b values	2	2	2	2 0-800-1,000	2

Abbreviations: DCE, dynamic contrast enhanced; DWI, diffusion-weighted imaging; FOV, field of view; HR, hazard ratio; HR-T_2_W-TSE, high resolution T_2_W-TSE; MRI, magnetic resonance imaging; T_1_W-SE, T_1_ weighted spin echo; T_2_W-TSE, T_2_ weighted turbo spin echo; VIBE, volume-interpolated breath-hold examination.

^a^ Repeat at least in one more orthogonal plane.

^b^ Acquire one measurement for the precontrast and approximately 12-measurement (to add up to 4 minutes) sequence with contrast as instructed.

^c^ Adjust to avoid wrapping or to obtain suitable coverage.
